# Free Riding on the Harm Principle

**DOI:** 10.1093/phe/phag012

**Published:** 2026-07-13

**Authors:** Lucie White

**Affiliations:** Department of Philosophy and Religious Studies, Utrecht University, The Netherlands

## Abstract

There are two main ways in which a mandatory vaccination policy is often defended—by accusing vaccine refusers of ‘free riding’, or by appealing to the ‘harm principle’. In their recent book, Roland Pierik and Marcel Verweij take the latter approach—an attractive option, as they note, due to the wide acceptance of the harm principle. But in arguing that vaccine refusal should indeed be thought to constitute harm, they end up endorsing a very controversial version of the harm principle, which we should not automatically expect to secure widespread support. Steven Smith suggests that this (common) argumentative strategy amounts to free riding on the reputation of the harm principle, and is an inevitable consequence of invoking the principle as the foundation of an argument. In its most basic form, the principle is hard to resist. But in order to use it to justify a policy, you need to make some assumptions about what constitutes harm, which will lose you supporters along the way. In appealing to the wide appeal of the harm principle, Pierik and Verweij do not adequately recognise the controversial nature of their claims, and thus the need for stronger and more elaborate arguments in their support.

Roland Pierik and Marcel Verweij (2024) provide us with a compelling and nuanced book-length argument for vaccine mandates. In Chapter 4, they lay out the ethical foundations of the argument. As they note, there are two main ways in which vaccine mandates are usually provided with an ethical foundation and justification. The first option: we could accuse vaccine refusers of unfair ‘free riding’, maintaining that they are benefitting from the public good of herd immunity while refusing to contribute to it, and that government intervention is justified to correct this injustice. With a clever argument, Pierik and Verweij dismiss this possibility. Though vaccine refusal may be free riding, they suggest, no-one can accuse vaccine refusers of behaving unfairly.

This argument is worth reading in full, but I focus here on the strategy that the authors do adopt: they suggest, instead, that vaccine mandates can be justified because vaccine refusal causes *harm to others,* and when conduct causes harm to others, government intervention is justified. This ‘harm principle’-based argument has been advanced (Flanigan, 2014; Brennan, 2018) and picked apart (Bernstein, 2017; Giubilini, 2020; White, 2024) before, and Pierik and Verweij acknowledge its limitations when used as the basis for a vaccine mandate in its familiar, direct form. So, they advance a novel twist on the argument, which warrants an in-depth exploration.

Pierik and Verweij find the inspiration for their understanding of the harm principle in the writings of its original advocate—John Stuart Mill. As they point out, Mill does not limit application of the harm principle only to direct harms: ‘he [also] invokes the principle as a basis for compelling members of society to contribute to collective projects that benefit all’ (2024, p.63). This includes, for example, ‘[giving] evidence in a court of justice, [bearing] his fair share in the common defence, and any other joint work *necessary to the interest of the society* of which he enjoys the protection’ (Mill qtd. in Pierik and Verweij, p.63, italics mine). ‘Protection against infectious diseases through the establishment of group-level protection’ (p.66), also known as ‘herd immunity’, is one such indispensable good, according to Pierik and Verweij.

Undermining the possibility of securing or maintaining a collectively-achieved good by refusing to contribute, particularly when that good is ‘indispensable for society’ or ‘necessary for societies to flourish’ (p.65) could be understood as doing harm. But here we run up against a familiar problem when using the harm principle to justify vaccine mandates. Can we really regard an individual as harming others through their failure to be vaccinated, when their individual conduct has a negligible effect on whether herd immunity is achieved? (cf. Brennan, 2018; Giubilini, 2020). Yes, say Pierik and Verweij, borrowing an argument from Elisabeth Cripps on collective harm: even where an individual’s contribution (or, for Pierik and Verweij, lack thereof) is not necessary to bring about a harm (see Cripps, 2011, p.177), individuals can be held collectively responsible as part of a harm-causing group, and ‘curtailment of their individual freedom is prima facie legitimate when the duties imposed on individuals are fairly allocated as part of a collective endeavour to prevent that harm’ (Pierik and Verweij, 2024, p.69). In addition, they point out, we should not underestimate the negative effects of non-compliance, which can make the refusal to contribute appear to be more attractive and legitimate to others.

So, we need a serious harm, threatening the ‘provision of services that are necessary for society to flourish’ (Pierik and Verweij, 2024, p.65), which can be avoided only through concerted, collective effort. Where these conditions obtain, we can compel individuals to contribute their fair share to this collective endeavour, even if their individual behaviour makes little difference to the ultimate outcome, and especially where non-compliance may encourage bad behaviour in others. These arguments have allowed Pierik and Verweij to avoid familiar objections to harm-principle-based arguments for mandatory vaccination. At the same time, we have, at this point, moved quite a distance from the usual, simple formulation of the harm principle. The principle we have now is, among other things, much more expansive. So perhaps we should now ask, what else would this particular harm principle justify (cf. John, 2021)?

Let’s explore just one example. Growing rates of obesity have introduced very significant costs into the healthcare system (Cawley *et al.*, 2021; Hecker *et al.*, 2022) and beyond (Hecker *et al.*, 2022). If obesity rates are to continue their upward trajectory, we will face a point at which the provision of a service, necessary to the functioning of society, i.e. an affordable, generally accessible and high quality healthcare system, is severely compromised. The costs of maintaining the system, at some point, will become prohibitive, and even if money was unlimited, other resources—time with healthcare professionals, hospital beds and equipment, surgeries performed, and so on, are necessarily in limited supply (Nath *et al.*, 2022). Increasing obesity-related disease burden, that is to say, is going to have a significant impact on the provision of health care services. In countries where prevalence is already very high, like the US, widespread obesity is also already affecting the provision of other necessary services, like the ability to field a combat-ready military (CDC, 2024).

Do we have, then, a prima facie case for government intervention? Mandated exercise programs, for example? Might we ban certain foods or place strict limits on their consumption? Mandate regular doctor’s visits to catch problems before they worsen and become more expensive and burdensome to treat? Even where an individual’s healthcare costs do not, on their own, break the bank, such restrictions might justifiably be imposed as part of an endeavour to avoid collective harm, and to allocate the duties to avoid that harm fairly. Pierik and Verweij emphasise that vaccine refusal can have encouraging, ripple effects on the conduct of others, but that too can be observed in the context of food consumption: a growing body of research indicates the profound effect that your food intake and choices have on the habits of those around you (see Robinson *et al.*, 2014; Higgs and Ruddock, 2020).^1,2^

One might argue that coercive government intervention is just what we need in this case. But even if this is true, it is clear that this would no longer be based on a harm principle upon which ‘every democrat, whether egalitarian, libertarian, communitarian, or socialist, must agree’ (2024, p.59)—Pierik and Verweij’s stated reason for opting for the principle in the first place. The harm principle we’ve ended up with is a controversial principle, which can countenance quite extensive interventions in our conduct. It’s also not clear that this is a reading that Mill would countenance—it makes the bright line Mill constantly draws between ‘conduct which affects only [oneself]’ and conduct which affects others (2016, p.22, see also p.21, 36, 37, 38, 42, 43, 48, 51, 52, and Smith, 2004) start to look quite blurry. If our choices about how to eat, how to exercise, and how to take care of ourselves belong to the sphere of other-regarding conduct that the government might justifiably intervene in, it becomes difficult to see where the self-regarding sphere of conduct—that Mill is so concerned with defending against outside interference with his harm principle—is to be located.^3^

Steven Smith (2004) suggests that the former problem is general and unavoidable when making use of the harm principle. In its most simple form—leave people alone when their conduct is not affecting others, intervene when they are causing harm to others—the principle seems hard to resist. But in order to use it to say anything at all, you need to make some assumptions about what constitutes harm, which will lose you some supporters along the way. If you continue to use the wide appeal of the harm principle as the basis for your argument, Smith argues, you are ‘free riding’ on the self-evident nature of its simple (yet empty) form, while failing to provide adequate justification for the more controversial claims that give substance to your argument.

This is not to say that we can’t make an argument for vaccine mandates—even one in which the avoidance of harm features as a chief consideration. But such arguments should not trade on the wide appeal of the harm principle. Invoking the harm principle does not abrogate—but can mask—the need for inevitably controversial arguments concerning *why* the conduct in question should be understood as harmful in a way that warrants government intervention—including what this means for other, similar cases, what kind of broader interventions this would warrant, and why these implications should be accepted. It also masks the fact that the answers to these questions do not only involve controversial assumptions about harm. We cannot answer them without thinking more broadly about what reasons we have to endorse, and refrain from, government intervention—about what we most value, prioritise, and what the appropriate role of government is.

It is difficult to withstand the rhetorical pull of an argument with which ‘every democrat…must agree’ (Pierik and Verweij, 2024, p.59). But to recognise that this policy will always be subject to disagreement is to recognise the importance of strong arguments in its favour, which engage the full range of contentious issues it bears upon.

## Notes

1 Particularly in the case of food consumption, we might have a stronger Millian basis, because it involves a collectively harmful *action* rather than a collectively harmful *omission.* Though, as Pierik and Verweij note, Mill does allow omissions to enter into the harm principle, he regards this as the exception, justified only in clear and grave cases (Mill, 2016; see also Pierik and Verweij, 2024, pp.62–3).

2 In addition, as an anonymous reviewer notes, including ‘setting a bad example’ within the purview of the harm principle also threatens to significantly broaden its scope.

3 Mill acknowledges, of course, that this line is necessarily blurred to a degree: ‘When I say only himself, I mean directly, and in the first instance: for whatever affects himself, may affect others through himself’ (2016, p.22)—still, he maintains most strenuously that ‘conduct which affects only [the person] himself’ in this sense is not a legitimate site of government intervention.
